# Vasoplegia: Mechanism and Management Following Cardiopulmonary Bypass

**DOI:** 10.5152/eurasianjmed.2022.20394

**Published:** 2022-02-01

**Authors:** Rizal Muhammad, Budi Baktijasa Dharmadjati, Eka Prasetya Budi Mulia, Dita Aulia Rachmi

**Affiliations:** Department of Cardiology and Vascular Medicine, Universitas Airlangga Faculty of Medicine, Dr. Soetomo General Hospital, Surabaya, Indonesia

**Keywords:** Cardiopulmonary bypass, methylene blue, nitric oxide, vasoplegia, vasopressin

## Abstract

Vasoplegia is defined by hypotension and low systemic vascular resistance despite the normal or elevated cardiac index, a complication frequently following cardiac surgery, carrying high morbidity and mortality rate. Vasoplegia is related with a profound systemic inflammatory response and is mainly mediated by cellular hyperpolarization, a relative vasopressin deficiency, and high levels of inducible nitric oxide, following cardiopulmonary bypass. Cardiopulmonary bypass is a distinct precipitant of vasoplegia, generally due to its association with nitric oxide production and severe vasopressin depletion. Postoperative vasoplegia is usually managed with vasopressors, of which catecholamines are the traditional agents of choice. Recent studies promote the use of non-catecholamine vasopressor (vasopressin) in restoring systemic vascular resistance. Alternative agents are also able to restore vascular tone and improve vasoplegia, including methylene blue, angiotensin II, hydroxocobalamin, and ascorbic acid; however, their effect on patient outcomes is still unclear.

## Main Points

- Currently, there is no guideline regarding vasoplegia syndrome.
- Vasoplegia is commonly defined by hypotension due to low systemic vascular resistance despite high cardiac index and aggressive therapy with fluids and vasopressors.
- Cardiopulmonary bypass is one of the most common risk factors of vasoplegia due to exposures of blood to foreign surfaces, leading to vascular disorders and vasodilation. Inflammation and impaired neurohumoral vascular tone regulation might also play a role.
- Non-catecholamine therapy showed promising efficacy in vasoplegia management, including methylene blue and angiotensin II.

## Introduction

Vasoplegia is a syndrome that is defined as abnormal/low systemic vascular resistance (SVR) manifesting as severe hypotension, with normal or elevated cardiac index. The causes of vasoplegia may vary, and several definitions are mentioned in the literature for specific causes and conditions. Also, there are variations in the used terminology. The absence of a clinical-based definition consensus restrains progress in understanding the pathophysiology of vasoplegia.^1^

Vasoplegia is a frequent complication following cardiac surgery, affecting up to 45% of the procedure. In general, the shock is limited in duration and degree of severity. However, some patients show severe vasoplegia, involving significant levels of morbidity and mortality.^2^ These patients suffer from decreased SVR and severe vasodilation which result in severe hypotension, even with normal cardiac index and adequate fluid resuscitation, leading to reduced tissue perfusion and metabolic acidosis.^1^ Up to date, there is no consensus regarding vasoplegia as a pathophysiologically distinct condition that represents the end stage of vascular homeostasis failure or as the final form of the vasodilation spectrum. It is assumed that the pathophysiology is similar to vasodilatory-induced septic shock, although the causative factors and several mediators seem to differ.

Until now, choices are quite limited, do not target the main pathophysiological pathways, and often require high-dose vasopressors to maintain adequate mean arterial pressure (MAP) following cardiac surgery. The typical first-line vasopressor therapy is catecholamines, and sometimes patients become refractory to vasopressors, called catecholamine-resistant hypotension, which results in high morbidity and mortality.^1,3^ Traditionally dealt with catecholamines and vasopressin arginine, vasoplegia that are refractory to these agents are very detrimental. Rescue therapy is urgently needed in refractive cases to limit prolonged hypoperfusion, maintain adequate organ function, and reduce morbidity and mortality. This review aimed to elaborate on the mechanism and management of vasoplegia, particularly following cardiopulmonary bypass (CPB).

### Vasoplegia

Vasoplegia is generally defined as severe refractive/persistent hypotension due to low SVR despite high cardiac index and aggressive therapy with fluids and vasopressors. There is no universal definition for vasoplegia. Several literatures mentioned that systemic inflammatory response to ischemia, surgical trauma, reperfusion, endotoxin release, or blood contact with the bypass circuit might lead to vasodilation. This vasodilation results in patient’s inability to maintain vascular tone resulting in inadequate end-organ perfusion.^4^

Most definitions of vasoplegia include the following elements:

- following CPB,
- low MAP (<50 mmHg),
- low pulmonary wedge pressure (<12 mmHg),
- low SVR (<800 dynes/s/cm^5^),
- normal or elevated cardiac index (CI) (>2.5 L/min), and
- refractory to fluid resuscitation and administration of high-dose vasopressors.

These criteria are relatively non-specific and are found, among others, in other disease states such as sepsis, adrenal insufficiency, and hepatic failure, with the etiology of shock being the distinction (infection and elevated blood lactate level in the case of sepsis and exposure to extracorporeal circulation in the case of vasoplegia).^5-7^

### Risk Factor

Vasoplegia has been reported in all age groups and in various clinical circumstances, such as sepsis, anaphylaxis (including protamine reactions), hemodialysis, hemorrhagic shock, and cardiac surgery. Depending on the definition and cohort of patients, the incidence of vasoplegia in the literature varies from 5% to 35%. It is even higher in patients undergoing ventricular support for end-stage heart failure (HF) or patients with left ventricular ejection fraction (EF) < 35%. Patients who experience vasodilation shock after heart surgery have the possibility of increased postoperative bleeding, kidney and liver injury, neurological dysfunction, and respiratory failure.^8,9^

### Drug Exposure

Blockade of the renin–angiotensin–aldosterone system (RAAS) is the central treatment for HF with decreased EF utilizing agents such as angiotensin-converting enzyme inhibitors and angiotensin receptor blockers, with or without renin–angiotensin–aldosterone system angiotensin neprilysin inhibitors. Several clinical trial demonstrated angiotensin blockade before cardiac surgery as an independent risk factor for perioperative vasoplegia (OR 11.9; 95% CI 2.7-53.1; *P* < .001) and inotropic therapy (OR 1.22; 95% CI 1.10-1.36; *P* < .001). A strong connection of angiotensin blockade with vasoplegia has led to the cessation of angiotensin blockers prior to heart surgery in order to maintain vascular tone.^8,10^

### Cardiac Surgery

Cardiopulmonary bypass usage differentiates cardiac surgery from other forms of surgery. It often exposes patients to a unique set of possible complications post CPB, including vasospasm, interactions of platelet–endothelial cell changes, and the overall inflammatory response caused by blood contact with synthetic surface of CPB systems. These result in low-flow microcirculation of the brain, heart, and other organs which can cause organ dysfunction.^8,11^

Cardiopulmonary bypass is a risk factor for patients experiencing vasoplegia, depending on the definition and type of heart surgery. Although the duration of vasoplegia can last up to 72 hours, poor hemodynamic during the initial post-CPB period lasting more than 36-48 hours is associated with an increased risk of systemic complications and mortality.^12^

Essential predictors of vasoplegia syndrome include type and technique of surgical procedure. Valve surgery and HF surgery (ventricular implantation; cardiac transplantation) significantly increase the risk of vasoplegia (valve-OR 1.52; 95% CI 1.66-1.99; *P* = .002; HF-OR 2.04; 95% CI 1.07-3.90; *P* = .031) compared with coronary artery bypass grafting.^3^

## Mechanism

### Vasodilatation

The pathophysiology of vasoplegic syndrome is similar to that of sepsis. Smooth muscle contraction, which is controlled by the involvement of various intrinsic and extrinsic pathways, results in systemic vascular tone. Receptors activation on the surface of vascular smooth muscle cells results from physiological vasoconstrictors such as norepinephrine and angiotensin II. The second messenger system was then activated by these receptors, which, in essence, through opening membrane-bound calcium channels increases intracellular calcium concentration. The complex developed in the cytosol between calcium and calmodulin contributes to the activation of kinases that, in turn, phosphorylate the light chain of myosin. Phosphorylation of myosin allows actin and myosin to interact and eventually contract muscles.^13^

Vasodilators lead to myosin dephosphorylation, such as atrial natriuretic peptide (ANP) and nitric oxide (NO), thereby restricting muscle contraction. The major factors responsible for decreased vascular tone are activation of the adenosine triphosphate-sensitive potassium channel (KATP channel) in the vascular smooth muscle plasma membrane, activation of the inducible form of nitric oxide synthase (NOS), and deficiency of vasopressin.^12^

From a cellular perspective, vasodilatory shock appears to be a complex process, but it is fundamentally a deficit in the vasoconstrictive ability of vascular smooth muscle. In general, the binding of angiotensin and catecholamine to receptors on the cell surface, followed by opening of voltage-gated calcium channels, will increase intracellular calcium concentration, resulting in contraction of the vascular smooth muscle cells. Increased concentrations of cytoplasmic calcium will create a stepwise reaction, in which calcium phosphorylate myosin successively catalyzing the cross-linking of the actin-myosin filament will result in muscle contraction and vasoconstriction.

Homeostasis of vascular tone is also regulated by vasodilation molecules, such as ANP or NO. This molecule triggers vasodilation through several mechanisms, which in turn increases intracellular concentrations of cyclic guanosine monophosphate (cGMP) leading to myosin phosphatase activation, myosin dephosphorylation, and vasodilation, counterbalancing the vasoconstriction process.^5^

**Figure 1.**
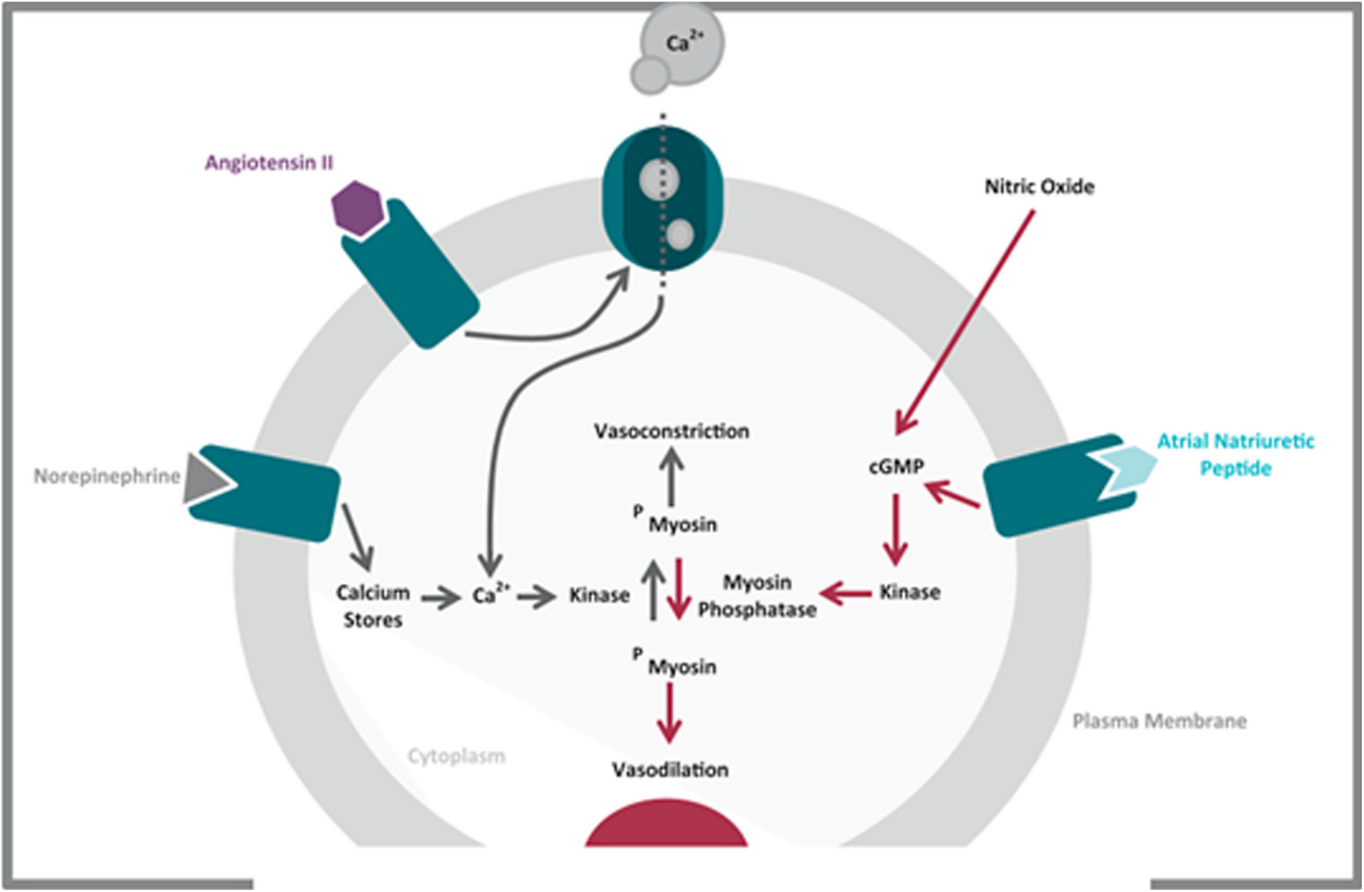
The cellular mechanism of vasodilatory shock. The smooth muscles of blood vessels contract when intracellular calcium levels rise and cause cross bonds between actin and myosin, which are phosphorylated. This process is triggered after vasoconstrictive mediators, such as angiotensin II or catecholamines, bind to surface receptors. Conversely, vasodilation occurs when molecules such as a nitric oxide or atrial natriuretic peptides produce an increase in intracellular cyclic guanosine monophosphate (cGMP) and deposition of myosin.^2^

Therefore, the vasoconstriction effect depends on calcium entry into the cytoplasm through the voltage-gated channel. In the setting of acidosis or adenosine triphosphate depletion, hyperpolarization of membrane will lead to inactivation of vascular gated calcium channels, a situation where vasoconstriction is not possible even if blood vessel cells are exposed to catecholamines in high dose. Other substances, including ANP, NO, and adenosine, can also cause prolonged opening and activation of the KATP channels. It is thought that this is an important physiological mechanism for neutralizing the transient period of local tissue ischemia. Nonetheless, this can be counter-productive if vasodilation prolongation causes low blood pressure and compromises the flow to vascular bed (Figure 1).^2^

Nitric oxide, which is involved as an activator for opening of KATP channel, is a very significant intercellular mediator of vasodilation shock. Nitric oxide is synthesized by a series of NOS enzymes, distinguished on the basis of organ location and basic activity. Nitric oxide synthase isoforms depend on calcium and are responsible for the production of constant low levels of NO, which are significant for interneuronal signals, regional blood flow regulation, and immunological modulation. Nitric oxide synthase isoforms that can be induced without calcium (iNOS) synthesize NO on demand and need several hours to react to physiological stress. This iNOS can trigger dysfunction of mitochondria, apoptosis, and multiorgan failure and is often involved as a mediator of distributive shock. However, it has an important physiological role and that may have to be considered a “necessary poison,” having both a direct and indirect protective and detrimental effect. Nitric oxide synthase induction, for example, is necessary for increasing myocardial NO levels, which promotes left ventricular relaxation and good diastolic filling.^2^

Inevitably, NO raises intracellular cGMP, subsequently reducing phosphorylation of myosin, deactivating calmodulin, and encouraging calcium-sensitive potassium efflux opening (KCA channels) to reduce vasoconstriction impact. The existence of NO, therefore, contributes to a condition of weakened contraction of the vascular smooth muscle.^12^

Vasopressin is an essential vasodilation modulator, in addition to membrane hyperpolarization and elevated NO concentrations. Relative vasopressin deficiency, which could be insufficient for the physiological stress severity following CPB, is consistent with prolonged shock. Initially, the serum concentration of vasopressin in acute hypotension is very high but eventually decreases to a sub-normal level. After prolonged stimulation of arterial baroreflex, this decrease is assumed to be triggered by the loss of neuro-hypophyseal deposits. This is mechanically essential since vasopressin deactivates the KATP channel directly, inhibits the rise in cGMP caused by NO (by binding to receptors of AVPR1), and decreases synthesis of NO. Vasopressin reduces the effect of hyperpolarization of the membrane, dephosphorylation of myosin, and accumulation of NO, which is an essential vascular tone modulator.^12^

### Cardiopulmonary Bypass

**Figure 2.**
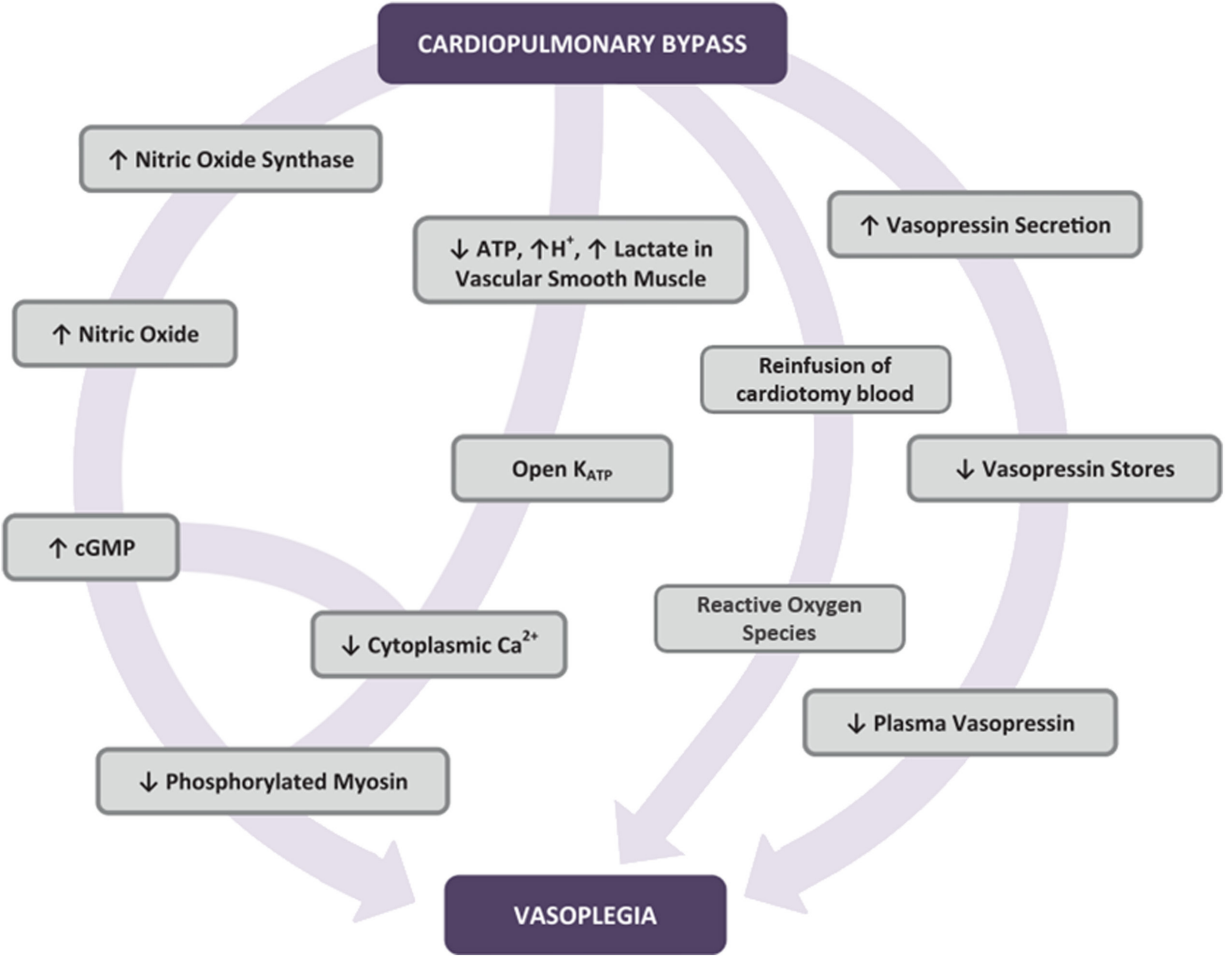
The mechanism of CPB-associated vasoplegia. Cardiopulmonary bypass triggers a severe inflammatory reaction that increases NO production, ATP depletion, and an increase in the vascular smooth muscle acidemia, which results in decreased myosin phosphorylation and vasodilation. Simultaneously, neuro-hypophyseal deposits from endogenous vasopressin are depleted rapidly, adding to the vasodilation effect and creating vasoplegia. ATP: adenosine triphosphate, cGMP: cyclic guanosine monophosphate, CPB: cardiopulmonary bypass; KATP: ATP-sensitive potassium channel.^2^

Considerable investigations into the mechanisms underlying vasoplegia have been carried out, mostly focusing on the physiological response to extracorporeal circulation. Cardiopulmonary bypass causes extensive secondary immunological responses due to cardiac and pulmonary ischemia–reperfusion injury, the release of endotoxins from the mucosal surface, and the cascade of complement activation after blood contact to the CPB circuit (Figure 2).^2^

Patients who undergo cardiac surgery with CPB have various exposures of blood to foreign surfaces, leading to vascular disorders and vasodilation. This involves baroreceptor stimulation and NO upregulation pathway due to intraoperative hypotension, activation of proinflammatory cytokines and complement from CPB equipment and blood re-transfusion, reperfusion injury due to the release of aortic cross-clamp, and increased NO regulation from the reversal of protamine coming out of pump.^3^

Reinfusion cardiotomy, hemolyzed blood containing activated substances, extraction of aortic cross clamps that can induce ischemia–reperfusion syndrome via activation of neutrophil, the secretion of reactive oxygen species, and direct damage to proteins, lipids, and nucleic acids are additional aspects that can lead to the severe inflammatory response during cardiac surgery. This promotes capillary permeability causing interstitial edema and reduces intravascular volume.^3^

The development of reperfusion injury involved oxidative stress, inflammation, calcium overload, and hypercontracture. Reperfusion injury begins as an ischemic event and is believed to end with the opening of mitochondrial permeability transition pore (mPTP). Griffiths and Halestrap^14^ proved that mPTP opening happened during reperfusion. When the blood flow restores to ischemic tissue, the respiratory chain is immediately exposed to oxygen. Reactive oxygen species increases due to a lower concentration of antioxidative agents in ischemic cells. Reactive oxygen species generates oxidative stress which later promotes endothelial dysfunction, DNA damage, and local inflammatory responses. These events may induce a cytokine storm, resulting in cell death caused by damage to cellular structures.^15,16^

Interestingly, the CPB contributes to an increase in the induced NO production, a concentration that correlates directly to the duration of the CPB. It has also been shown that elevated serum concentrations of these molecules associate with the occurrence of systemic inflammatory response syndromes, promoting the theory that, at least in part, post-CPB vasoplegia is an inflammatory response. This downstream consequences of inflammatory and vasoactive agents cause disturbances of reactivity and initial vascular tone.^2^

However, the only cause of post-CPB vasoplegia is not a general inflammatory response with elevated production of NO. Impaired neurohumoral vascular tone regulation in the context of vasopressin deficiency has an important role to play. It is understood that serum vasopressin levels increase during CPB and remain high or normal in patients who do not experience post-CPB vasoplegia. Meanwhile, the serum concentration of vasopressin in patients who experienced vasoplegia after CPB was found to be very low, indicating a chronic decrease in neuro-hypophyseal vasopressin storage. This relative reduction in vasopressin indicates that CPB is a very stressful trigger in susceptible patients and can cause severe vasoplegia.^12^

### Management

Initial management of patients with vasoplegia using vasopressors restores hemodynamic function in most patients but not in all patients; The most commonly used vasopressors are norepinephrine, vasopressin, and phenylephrine. Most of the treatment options used in septic shock have been extrapolated for use in vasoplegic syndrome due to the similarity between vasoplegic syndrome and sepsis, along with a lack of supporting evidence.^6^ A review of the literature available on vasoplegia therapy shows that norepinephrine, phenylephrine, vasopressin, methylene blue, and angiotensin II can all increase the MAP after CPB (Table 1). There is not enough evidence to suggest the superiority of certain vasopressors from other available drugs, both physiologically or clinically. It is recommended that when target pressure cannot be achieved by a single dose infusion of a single drug, a second drug with a different mechanism of action should be used. The side effect profiles of these various drugs appear to be similar in the available studies, so initial therapy usually involves volume expansion and administration of norepinephrine.^17^ Vasoactive drugs in vasoplegia management were summarized in Table 1.

### Volume Expansion and Blood Products

Although arterial baroreceptors are the most sensitive receptors, low-pressure receptors in the heart and vena cava called cardiopulmonary receptors also play a role in hemodynamic modulation. They respond mainly not only to changes in volume but also chemical stimuli. They project through the vagal afferents and spinal sympathetic afferents into the spinal cord so that vasodilation occurs and inhibits vasopressin release.^13^

A significant aspect of early therapy for postoperative vasoplegia is to recognize fluid responsiveness. Because hypovolemic shock is related to perioperative bleeding, it can occur together with vasoplegia; a wise blood product transfusion must be used to correct severe anemia. In general, a restrictive transfusion strategy is favored over a more liberal strategy. However, unnecessarily vigorous (exceeding 20-30 mL/kg) fluid resuscitation causes undue vascular pressure and shear stress, needless raises in heart filling pressure, and dangerous accumulation of pulmonary extravascular fluid. Finally, excessive fluid administration in pure vasodilatory shock is related to increased mortality and should be avoided.^2^

### Catecholamine

Catecholamines provide physiological effects by stimulation of the adrenergic receptors. It has traditionally been the agent of choice for vasodilation shock therapy. Norepinephrine, epinephrine, and dopamine have all been successfully used without restricting end-organ perfusion to improve MAP. While there are various vasopressors to support the vascular tone, norepinephrine is recommended in patients with distributive shock.^2,6^

There are 2 types of alpha-adrenergic receptors (alpha-1 and alpha-2). Located in vascular smooth muscle sarcolemma are alpha-1 vasoconstrictor receptors, while those located in terminal varicose veins are alpha-adrenergic receptors that provide feedback to inhibit the release of norepinephrine. Pharmacologically, alpha-adrenergic receptors mediate responses where the effects mimic the effects of phenylephrine pharmacological agents. Among catecholamines, the relative potential of alpha1 agonists is norepinephrine > epinephrine > isoproterenol. Physiologically, norepinephrine released from nerve terminals is the main stimulus for vascular alpha1-adrenergic activity.^13^

Compared with other catecholamines, the study of norepinephrine showed a benefit in mortality and was the preferred first-line agent, especially in septic shock. Studies comparing norepinephrine with different catecholamines combinations, such as epinephrine or dobutamine, however have failed to prove a definite benefit. On the other hand, in randomized controlled trials (RCTs), dopamine carries an elevated risk of arrhythmia and mortality compared to norepinephrine, so it should not be used as a first-line drug. The risk for serious peripheral vasoconstriction and end-organ damage are common concerns about the use of high-dose vasopressors. The requirement for high-dose catecholamines should, however, encourage physicians to consider transitioning to non-catecholaminergic agents or adding them.^19^

### Vasopressin

Increased levels of the pituitary hormone arginine vasopressin have contributed to the increase in SVR.^13^ Vasopressin binds to V1 receptors, which is important for increasing vasoconstriction by inhibiting the opening of KATP channels and reducing NO production, providing a mechanism that is entirely catecholamine free.^19^ In septic shock, vasopressin plasma concentrations increase in the early stages of shock, but after 24 hours, it drops to sub-normal levels, which may be a mechanism for loss of vascular tone.^1^

The use of non-catecholaminergic agents for vasodilation shock therapy can overcome several difficulties when dealing with severe vasoplegia, including membrane hyperpolarization and associated catecholamine resistance. Non-catecholamine agents can also provide a synergistic effect and make it possible to reduce the dosage of 1 particular agent, creating a more balanced approach to vasopressor therapy. This may be especially important with vasopressin, the use of which is biochemically supported by the presence of vasopressin deficiency after CPB.^12^

In a recent Vasopressin versus Norepinephrine in Patients with Vasoplegic Shock after Cardiac Surgery (VANCS) study, Hajjar et al^17^ randomly allocated patients with post-CPB vasoplegia to vasopressin or norepinephrine as the primary agent, showing a significant reduction in the composite endpoint of 30-day mortality or postoperative complications, which is supported almost exclusively by decreasing the occurrence of acute renal failure (OR 0.26; 95% CI 0.15-0.46; *P* < .001). Atrial fibrillation, a general supraventricular arrhythmia after cardiac surgery, was significantly less frequent in patients receiving vasopressin compared with patients receiving norepinephrine (OR 0.37; 95% CI 0.22-0.64; *P* = .004), which describes the inflammatory response in addition to increased stimulation of beta-1 receptors by norepinephrine in the atrium, directly involved in its occurrence after cardiac surgery.

The VANCS trial also compared vasopressin to norepinephrine for vasoplegic syndrome after cardiac surgery. Vasopressin was superior to norepinephrine as it showed lower rates (32% and 49%, respectively) of all-cause mortality and severe complication within 30 days (unadjusted hazard ratio 0.55; 95% CI 0.38-0.80; *P* = .0014). Hence, it suggests vasopressin be used as the first-line therapy. However, it is important to note that 53% of patients in this trial had normal preoperative EF (EF greater than 60%).^17^ Although the authors indicated that the cardiac index was not affected after vasopressor infusion, the cardiac index is predicted to decrease in patients with decreased EF after pure vasoconstrictor infusion.^20,21^ Myocardial dysfunction associated with vasopressin does not result from an increase in SVR but from a direct effect on cardiac contractility.^22^ It can be detrimental to maintain systemic blood pressure only by increasing SVR without increasing cardiac contractility, and a decrease in SVR may be associated with improvement in cardiac index.^23,24^ Therefore, our target should be to maintain SVR within normal limits.^25^ We believe it is arguable whether the same agent has the same superiority for patients with reduced EF. We proposed taking into consideration the patient’s left ventricular function to decide the appropriate agent. Contrary to vasopressin, its receptor antagonist has been demonstrated to improve left ventricular systolic function.^20,26^

In patients undergoing cardiac surgery, another interesting property of vasopressin is its neutral effect on heart rate and myocardial oxygen intake. In this case, vasopressin therapy as an additional agent will decrease the total dose of catecholamine needed for systemic vascular tone recovery and reduce the risk of atrial fibrillation.^19^

### Methylene Blue

Among the non-catecholamine options for the management of vasoplegia, methylene blue (MTB) is increasingly popular. Methylene blue is an odorless, water-soluble, blue-green crystalline powder that turns blue every time it is mixed in solution. Therapeutic boluses 1-2 mg/kg is usually given for 10-20 minutes, or up to 1 hour, for vasodilation shock. Intravenous administration usually has a terminal half-life of 5-6 hours, and a continuous infusion of 1 mg/kg/hour can be beneficial after an initial bolus of up to 48-72 hours without compromise in splanchnic perfusion.^27^

More suppression of vasodilation than overt vasoconstriction is involved in the mechanism of action of MTB. Methylene blue decreases the production of NO through inhibition of NOS and further activation of soluble guanylate cyclase (sGC).^28^ Methylene blue inhibits the aggregation of cGMP by binding to sGC and inducing enzyme inhibition, and directly competing with NO to activate sGC. The main consideration for the administration of MTB for this indication is the unknown effect of a further increase in pulmonary vascular resistance after inhibition of NO and sGC, which has the potential to cause right HF.^29^

Methylene blue rescue therapy can recover blood vessel tone and can improve clinical outcomes, including mortality and length of stay.^2^ Levin et al^12^ described a decrease in mortality (0 vs. 21.4%, *P* = .01, n = 56) and duration of vasoplegia (2 hours vs. 48 hours; *P* = .002) of patients randomized to receive MTB versus placebo in patients with vasoplegia at the postoperative period of the heart.

Although adverse reactions such as serotonin syndrome, hemolytic anemia in glucose-6-phosphate-dehydrogenase (G6PD) deficiency patients, and hypoxia arising from pulmonary vasoconstriction inhibition can occur caused by this treatment, MTB must be carefully regarded under conditions of selective serotonin reuptake inhibitor (SSRI) treatment for depression, since severe serotonin syndrome will greatly complicate post-cardiac rehabilitation in adult heart surgery.^29^ With the MTB side effect profile, it is necessary to carry out a continuous search for the possibility of additional non-catecholamine therapy in cardiac surgery-associated vasoplegia.

In conclusion, the concepts of MTB in treating vasoplegia were: (1) MTB is safe in the recommended doses (the lethal dose is 40 mg/kg); (2) MTB does not cause endothelial dysfunction; (3) the MTB effect appears in cases of NO upregulation; (4) MTB is not a vasoconstrictor; by blocking the cGMP pathway, it releases the cAMP pathway, facilitating the norepinephrine vasoconstrictor effect; (5) the most used dosage is 2 mg/kg as IV bolus, followed by the same continuous infusion because plasma concentrations sharply decrease in the first 40 minutes; and 6) there is a possible “window of opportunity” for MTB's effectiveness.^27^

However, the major challenges of MTB usage are: (1) observations about side effects; (2) the need for prophylactic and therapeutic guidelines, and (3) the need for the establishment of the MTB therapeutic window in humans. Methylene blue action to treat vasoplegic syndrome is time-dependent. Therefore, the great challenge is the need for the establishment of the MTB therapeutic window in humans.^27^

### Angiotensin II

Angiotensin II is generated in healthy individuals by the division of angiotensin I by ACE in response to hypotension, which has a global effect on systemically located type 1 angiotensin receptors in many locations. Angiotensin II acts via many pathways, such as systemic and renal arteriolar vasoconstriction, increasing sympathetic activity, stimulating the release of endogenous vasopressin from the posterior pituitary gland, and the release of aldosterone from the adrenal gland. This stimulation produces direct arterial and venous vasoconstriction in the vascular smooth muscle, as well as an improvement in MAP regardless of adrenergic stimulation, in addition to improved vascular permeability.^13,30^

When hypotension occurs, human physiology adopts the RAAS system. The reason is based on the potential benefits of imitating more closely the natural physiological response to shock, which includes increased secretion of catecholamines, vasopressin, and the RAAS hormone.^31^

While catecholamines are known to narrow mesenteric blood vessels, angiotensin II is considered capable of mobilizing venous blood from the mesenteric circulation, also increasing preload and promoting lactate clearance.^32^

Recent RCT on synthetic human angiotensin II has emerged in this setting as an important rescue agent because it can recover systemic vascular tone to catecholamine-resistant vasodilation shock (OR 7.95; 95% CI 4.76-13.3; *P* < .001). Angiotensin II also significantly improved survival (adjusted HR 0.44; 95 % CI 0.24-0.80; *P* = .007) and the need for renal replacement therapy in distributive shock treatment.^31^

### Hydroxocobalamin (vitamin B12)

Vitamin B12 is another therapeutic agent that receives a lot of attention for saving hemodynamics in vasoplegia. Vitamin B12 is approved by the US Food and Drug Administration for the treatment of cyanide poisoning and carbon monoxide poisoning (through inhalation of smoke). Vitamin B12 can increase MAP in some cases where MTB therapy has not been successful and has recently been used off-label to treat vasoplegia in post-CPB patients and liver transplant patients.^4,30^

Vitamin B12 acts as a NO sponge by cleaning NO, spreading freely, and effectively scavenging NO, which is released in endothelial cells either in blood vessels or diffuses into the perivascular space. In addition to scavenging free NO, vitamin B12 dampens NO signals, reducing vasodilation by mode of oxygenation.^33^ Cobalamin can also act as a catalyst in the presence of O_2_ and vitamin C, which recently reported being applied in vasoplegia to inactivate or suppress NO signaling.^34^

Vascular mechanisms of action for vitamin B12 may include inhibition of NO production and increased vasodilatory agents clearance such as hydrogen sulfide and endothelium hyperpolarization factors. A cohort retrospective study has suggested that additional therapy of vitamin B12 can improve hemodynamic responses to MTB significantly.^35^

The optimal dose and time of hydroxocobalamin for vasodilatory shock is unknown. Several case studies report bolus doses of 5 g for 10-15 minutes per day, as recommended by the manufacturer for cyanide toxicity, and can be repeated up to 48 hours after cardiac surgery. In general, MTB and vitamin B12 can be used as a last resort.^33^

Vitamin B12 therapy can also be an alternative agent for saving severe vasoplegia when there are strong contraindications for MTB, such as G6PD deficiency or concomitant treatment with SSRI. Although vitamin B12 extends the therapeutic armamentarium for vasoplegia, it cannot be separated from the side effect profiles such as cobalt toxicity due to repeated doses in patients with kidney failure. Dramatic hemodynamic response to vitamin B12 requires additional trials to evaluate what appears to be a promising option for the management of vasoplegia.^4^

### Ascorbic Acid (vitamin C)

Vitamin C is the second micronutrient that begins to emerge in the role of rescue therapy for vasoplegia. This is an important cofactor in the endogenous catecholamines biosynthesis. Humans cannot synthesize vitamin C endogenously and need food supplementation to maintain adequate concentration.^6^

Catecholamines are endogenously synthesized in the adrenal medulla of healthy individuals. In the biosynthesis of dopamine and norepinephrine, vitamin C is a cofactor for dopamine β-hydroxylase and tyrosine hydroxylase. However, hormone production was found to be suppressed in the critical disease setting.^6,36^

Humans cannot synthesize vitamin C endogenously. Because of its water solubility, CPB has been shown to eliminate vitamin C from the blood, thereby reducing the concentration of vitamin C after bypass. In theory, exogenous administration can increase adrenal deposits, thereby increasing catecholamine production through this biochemical pathway. In addition, vitamin C has been proposed to improve microcirculation, scavenge ROS, reduce NOS induction, and increase sensitivity to catecholamines by reducing adrenergic receptors back to baseline conditions.^34^

Therefore, high doses of vitamin C therapy may significantly restore systemic vascular tone to reduce the need for vasopressors within 24 hours and have minimal or unknown side effects in cardiac surgery populations.^34^

### Corticosteroids

Corticosteroids are often used to treat vasodilatory shock, assuming that in critical illness, they can complement a depleted adrenal axis.^30^ Corticosteroids alone can help restore blood pressure in vasoplegic syndrome, although studies have focused primarily on the septic shock population.^6,37,38^ Experimental studies have shown restoration of vascular responsiveness to vasopressors, likely through a non-genomic inhibition of the arachidonic acid cascade and a genomic inhibition of the nuclear translocation of the NF-κB transcription factor.^39^ In addition, glucocorticoids also inhibit iNOS and COX2 synthesis.^40,41^ Finally, with an improvement in alpha-adrenergic receptor gene expression, low doses of glucocorticoids seem to restore vascular responsiveness to norepinephrine.^18,42^

At this point in time, however, few clinical trials have specifically evaluated the use of corticosteroids for the treatment of vasoplegic syndrome. Smaller studies have previously shown a decrease in CPB-associated inflammatory response.^43^ More recently, 2 larger clinical trials showed no benefit from the intraoperative use of methylprednisolone or dexamethasone, but the results evaluated were not specific to the response of blood pressure or vasoplegic syndrome.^44,45^ Prophylactic dexamethasone administration to patients undergoing cardiac surgery may decrease the composite outcome of death and other major morbidities.^44^ The use of corticosteroids for the treatment of vasoplegia following CPB has not been well studied, but their adverse effects, including delayed wound healing, hyperglycemia, and an increased risk of gastrointestinal bleeding, should be closely considered in this population.^2^ However, the 200 mg daily dose of hydrocortisone may be reasonable in patients requiring prolonged vasopressor doses to address any adrenal insufficiency concerns or in the case of refractory septic shock.^6,46^

## Conclusion

A growing priority for the uniform definition of vasoplegia syndrome is becoming very important to direct future research in this field. Cardiac intensivist must know important risk factors before surgery that can influence patients to vasoplegia syndrome, in addition to factors that worsen the condition, such as hypoxia, acidosis, and electrolyte disorders. The multifactorial etiology of vasoplegia after cardiac surgery requires a broad diagnostic and management approach. To date, only 2 vasopressors classes are available: catecholamines and vasopressin, in which both classes have a narrow therapeutic window because of substantial toxic effects at high doses. Targeting therapy in the mechanism of endogenous NO release is an essential therapeutic approach for rescue agents, including vitamin B12 and MTB. Vitamin C is hypothesized as a catalyst in catecholamine biosynthesis. It should be noted that some non-catecholamines agents used to treat vasoplegia, such as MTB, angiotensin II, vitamin B12, or vitamin C, have not been studied well in RCT. However, available data obtained from mechanistic and observational studies are promising.

## Figures and Tables

**Table 1.** The vasoactive drug in the management of Vasoplegia.2,13

	**Recommended dose**	**Mode of action**	**Advantage**	**Disadvantage**	**Evidence**
Catecholamines					
Norepinephrine	0.01-0.1 mcg / kg / minute continuous infusion	Significant α1, α2 agonism. Moderate β1 agonism.	Improving MAP mainly through increased SVR but can also provide inotropic support	High doses may be needed to achieve hemodynamic goals in severe vasoplegia	Recommended as a first-line agent based on septic shock RCTs. Can have a mortality benefit compared to other catecholamines used in isolation.
Epinephrine	0.01-0.5 mcg / kg / minute continuous infusion	Significant α1, α2 agonism. Significant β1 agonism.	Increases MAP and provides inotropic support	Few studies focus on examining their use as first-line agents for vasodilatory shock	Proportional efficacy compared to the combination of norepinephrine and dobutamine when vasopressor and inotropic are required
Dopamine	0-20 mcg / kg / minute continuous infusion	Dose-dependent adrenergic agonism. α1 agonism as dose increases.	Increased SVR and inotropy are dose-dependent	Increased risk of arrhythmias compared with other catecholamines	An RCT meta-analysis showed an increased risk of death compared to norepinephrine
Non-catecholamines					
Vasopressin	1.2-6.0 units / h continuous infusion	Repletion of vasopressin in ADH depleted state. V1 agonism.	Reducing catecholamines requirement to achieve the MAP target. Can reduce the severity of kidney failure.	As a first-line agent, there is no significant mortality benefit compared to norepinephrine	Use is supported by several RCTs and observations of post-CPB severe vasopressin deficiency
Methylene Blue	1.5-2 mg / kg bolus	Inhibition of guanylyl cyclase and inducible endothelial NO synthase	A single bolus can increase MAP rapidly in severe vasoplegia	Can trigger serotonin syndrome, hemolytic anemia and interfere with oximetry	There is no high-quality RCT investigating its use Retrospectively related to mortality benefit if given early in vasoplegia
Angiotensin II	Start 20 ng / kg / min, continuous infusion	AT1 agonism. Stimulation of aldosterone release. Increase in ADH synthesis.	Able to increase MAP dramatically and reduce the need for catecholamines	Limited data. May interfere with endogenous vasopressin synthesis.	One recent RCT suggested an increase in hemodynamics compared to placebo
Vitamin B12	5 g infusion over 5 min	Inhibition of NO directly and inducible endothelial NO synthase. Inhibition of hydrogen sulfide.	Increase MAP and avoid some of the risks associated with methylene blue	Not properly investigated	Only described in case reports
Vitamin C	6 g intravenous bolus per day	Cofactor for catecholamine synthesis.	Can accelerate shock reversal when combined with vitamin B12	Limited data on safety and efficacy	One recent retrospective study suggested the benefits of hemodynamics and mortality in septic shock patients

Abbreviation, ADH: antidiuretic hormone; CPB: cardiopulmonary bypass; MAP: mean arterial pressure; NO: nitric oxide; RCT: randomized controlled trial; SVR: systemic vascular resistance.
